# *Platycodon grandiflorus* Root Extract Improves Learning and Memory by Enhancing Synaptogenesis in Mice Hippocampus

**DOI:** 10.3390/nu9070794

**Published:** 2017-07-23

**Authors:** Jin-il Kim, Seong Gak Jeon, Kyoung Ah Kim, Jwa-Jin Kim, Eun Ji Song, Yukyoung Jeon, Eunbin Kim, Kyung Bok Lee, Jong Hwan Kwak, Minho Moon

**Affiliations:** 1 Department of Nursing, College of Nursing, Jeju National University, Jeju-si 63243, Korea; neoreva@hanmail.net; 2 Department of Biochemistry, College of Medicine, Konyang University, Daejeon 35365, Korea; jsg7394@naver.com (S.G.J.); helloimka@naver.com (K.A.K.); thddmswl1028@gmail.com (E.J.S.); kyunglee@konyang.ac.kr (K.B.L.); 3 Department of Biomedical Science, Jungwon University, Goesan-gun, Chungbuk 28024, Korea; kjj1021@naver.com; 4 Department of Anatomy, School of Medicine, Chungnam National University, Daejeon 34134, Korea; 5 LES Corporation Inc., 4 Munhwawon-ro 46beon-gil Yuseong-gu, Daejeon 34167, Korea; 6 School of Pharmacy, Sungkyunkwan University, Suwon-si, Gyeonggi-do 16419, Korea; kisara1203@hanmail.net (Y.J.); eunbin.kim423@gmail.com (E.K.)

**Keywords:** *Platycodon grandiflorus*, platycodin D, cognition, synaptogenesis, neuritogenesis

## Abstract

*Platycodon grandiflorus* (Jacq.) A.DC. (PG) has long been used as an ingredient of foods and is known to have beneficial effects on cognitive functions as well. The present study examined the effect of each PG extract (PGE) from root, aerial part, and seeds on cognitive functions in mice. Changes in spatial learning and memory using a Y-maze test, and markers of adult hippocampal neurogenesis and synaptogenesis were examined. Moreover, changes in neuritogenesis and activation of the ERK1/2 pathway were investigated. Results indicated that mice administered PGE (root) showed increased spontaneous alternation in the Y-maze test and synaptogenesis in the hippocampus. In addition, PGE (root) and platycodin D, the major bioactive compound from the PG root, significantly stimulated neuritic outgrowth by phosphorylation of the ERK1/2 signaling pathway in vitro. These results indicate that the PGE (root), containing platycodin D, enhances cognitive function through synaptogenesis via activation of the ERK1/2 signaling pathway.

## 1. Introduction

Hippocampal synaptogenesis, the formation of synapses between neurons in the hippocampus and adult hippocampal neurogenesis, and the generation of new neurons from neural stem cells in the dentate gyrus are key phenomena for cognitive function. Numerous studies have shown the relationship between hippocampal synaptogenesis and learning and memory in mice [1,2], as well as other animals, such as rats [3,4] and birds [5]. Therefore, abundant studies have attempted to enhance the synaptogenesis of hippocampal neurons by various interventions, including enriched environment [1], exercise [6], hormones [7,8,9], and natural plant materials [10,11]. In addition to synaptogenesis, it has been shown that adult hippocampal neurogenesis is associated with learning and memory, such as long- and short-term spatial memory [12]. An increasing number of studies have been conducted to stimulate adult hippocampal neurogenesis using diverse approaches, such as hormones [13,14], pharmaceutical approaches [15], exercise [16,17], enriched environment [18], as well as natural plant materials [11,19].

Synapse formation or neurogenesis requires interactions between complex signaling molecules. Since a number of signaling pathways mediate synaptogenesis, by either activation or inhibition, the induction of an appropriate signaling pathway is critical for the activation of synaptogenesis and neurogenesis. Amongst the signaling pathways, the mitogen-activated protein kinase/extracellular signal-regulated kinase (MAPK/ERK) pathway has an important role in synapse formation and adult hippocampal neurogenesis. Many studies have reported that the MAPK/ERK pathway is involved in the formation of functional synapse and neurite growth [20,21], induction of *N*-methyl-D-aspartate (NMDA) receptor-dependent long-term potentiation [22], and the generation of synaptogenic neurotropic factors [23,24,25]. In accordance with the relationship between the MAPK/ERK pathway and adult hippocampal neurogenesis, the MAPK/ERK pathway is known to be involved in the generation of hippocampal neurons as well. The MAPK/ERK pathway has been shown to involve the hippocampal neurogenesis in studies examining interventions to promote adult hippocampal neurogenesis, such as administration of valproate [26], vanadium compounds [21], Ginsenoside Rd [27], and amodiaquine [15]. Therefore, an intervention that induces the MAPK/ERK pathway might be an effective strategy to improve cognitive function via increased synaptogenesis and adult hippocampal neurogenesis.

*Platycodon grandiflorus* (Jacq.) A.DC. (PG) is commonly consumed as an ingredient of foods, such as salad, soup [28], seasoned mixed greens [29], and sauces [30] in Asia. In oriental traditional medicine, it is considered as a therapeutic constituent of medicines for amnesia and dementia, as well as inflammatory diseases [28,31,32,33]. Studies using the root of PG have demonstrated that PG attenuates lipopolysaccharide-induced inflammatory responses in microglial cells [34] and increases adult hippocampal neurogenesis by inducing neuronal cell proliferation in middle-aged mice [19]. Studies using specific components extracted from the root of PG have reported that triterpenes contained in the root of PG have protective effects against brain injury [28,35,36] and cognitive enhancing effects [37,38,39,40]. In particular, oral administration of triterpenes extracted from PG ameliorated learning and memory performances in behavioral tests in animals with memory impairment induced by scopolamine [39,40], ethanol [38], and amyloid β-protein (Aβ) [37]. Additionally, the major chemical constituents that mediate the therapeutic effects of PG have been reported to be triterpenoid saponins composed of platycoside, platycodin, and platycogalacin [28,41]. Interestingly, studies have reported that triterpenoid saponins from PG activate the MAPK/ERK pathway in osteoblasts [42] and human endothelial cells [42]. However, few studies have examined whether triterpenoid saponins from PG have effects on cognitive function in mice and on the MAPK/ERK pathway in neuronal cells. PG consists of three structural parts, including the roots, aerial part, and seeds, and each structure contains bioactive ingredients with different chemical compounds, respectively [43,44,45]. Thus, it can be speculated that the roots, aerial part, and seeds might have different effects on learning and memory. Indeed, no studies have uncovered the memory-enhancing effects of PG using behavioral tests, nor compared the effects of each structural part of PG (roots, aerial part, and seeds) on cognitive functions.

Therefore, to expand our knowledge about the effects of PG on cognitive function and their mechanisms, we explored the histological, behavioral changes in mice, as well as its molecular mechanisms following the administration of PG extract (PGE). This study demonstrated that the root extract of PG improves learning and memory by enhancing synaptogenesis induced by the activation of the MAPK/ERK signaling pathway.

## 2. Materials and Methods

### 2.1. Materials and Reagents

The PC12 cell line (rat adrenal pheochromocytoma cell line) was purchased from the Korean Cell Line Bank (Seoul, Korea). C57Bl/6 mice were purchased from Koatech (Pyeongtaek, Gyeonggi-do, Korea). A cryostat microtome was purchased from Leica Biosystems (Wetzlar, Hesse, Germany). Goat anti-doublecortin (DCX) antibody (1:1000) was purchased from Santa Cruz Biotechnology (Dallas, TX, USA). Biotinylated horse anti-goat immunoglobulin (Ig) G (1:200) was purchased from Vector Laboratories Inc. (Burlingame, CA, USA). Synaptophysin and β-actin were purchased from Sigma-Aldrich (St. Louis, MO, USA). 

### 2.2. Plant Material

Roots, aerial parts and seeds of PG were obtained from a farm (Bongsan-myeon, Yesan-gun, Chungcheongnam-do, Korea) in October 2014. Each of voucher specimens (root: SKKU-Ph-14-011, aerial part: SKKU-Ph-14-012, and seeds: SKKU-Ph-14-013) was deposited in the School of Pharmacy, Sungkyunkwan University.

### 2.3. Extraction from Root, Aerial Parts, and Seeds of PG

Extractions from each part of PG were prepared as described previously [46]. The roots and aerial parts of PG were cut into small pieces, and each of the specimens was lyophilized at −50 °C for 48 h. The dried roots (1.0 kg), aerial parts (0.82 kg) and seeds (2.0 kg) were extracted twice with ethanol (EtOH) at room temperature, and once with EtOH at 60 °C. Each of the EtOH extracts of PG (PGE) was concentrated under reduced pressure to give root (77.7 g), aerial part (56.0 g), and seed (48.3 g) extracts.

### 2.4. Isolation of Platycodin D and Platycodin D2 

The root extract (PGE (root), 70 g) was suspended in distilled water (700 mL). The resulting solution was consecutively partitioned with dichloromethane (CH_2_Cl_2_), ethyl acetate (EtOAc), and *n*-butanol (*n*-BuOH) to yield CH_2_Cl_2_ (2.52 g), EtOAc (2.77 g), *n*-BuOH (13.01 g), and H_2_O (50.12 g) fractions. The butanol fraction was fractionated on a silica gel column using a stepwise elution with CH_2_Cl_2_-Methanol (MeOH)-H_2_O (40:10:1, 70:30:3, 60:40:4, and 60:50:10) to give seven subfractions (D-1 to D-7). Subfraction D-5 was chromatographed on silica gel with EtOAc-MeOH-H_2_O (25:5:4), and a selected fraction was rechromatographed on RP-C18 column (50% MeOH in H_2_O) and Sephadex LH-20 column (MeOH) to obtain platycodin D (7.3 mg). Subfraction D-6 was subjected subsequently to silica gel (EtOAc/MeOH/H_2_O, 20:5:4), RP-C18 (48% MeOH in H_2_O), and Sephadex LH-20 (MeOH) column chromatography to afford platycodin D2 (8.8 mg). The purities of platycodin D and platycodin D2 were determined to be above 97.5% by high-performance liquid chromatography (HPLC) analysis with refractive index (RI) detection.

### 2.5. HPLC Analysis of the PGE (Root)

The HPLC analysis was performed as described previously [46]. HPCL analysis was conducted with a Knauer Smartline system (a Manager 5000, two Pump 1000 and UV Detector 2500) equipped with a phenomenex Kinetex 5 µ C18 100 A column (150 × 4.6 mm) using a gradient elution. The eluent consisted of acetonitrile (A) and 0.5% phosphoric acid in water (B). The gradient profile was: 0–20 min, isocratic 20% A in B; 20–40 min, linear change from 20% to 30% A in B; 40–50 min, isocratic 30% A in B. The flow rate and column oven temperature were set at 1 mL/min and 40 °C, respectively. The UV absorption was measured at a wavelength of 210 nm. The solution of extract was prepared by dissolving 25 mg of the root extract in 1 mL of MeOH, and standard working solutions of platycodin D and platycodin D2 were prepared by serial dilution with MeOH, yielding concentrations of 500, 200, 100, 50, and 20 μg/mL, respectively.

### 2.6. Animals and PGE Administration

**Figure 1:**
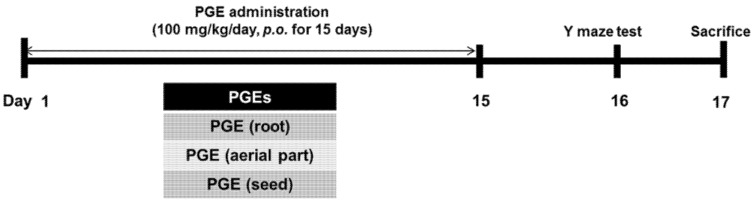
Oral administration of PGEs (100 mg/kg/day) for 15 days. PGEs are classified into three different parts including PGE (root), PGE (aerial part), and PGE (seed). The cognition of mice treated with three different PGEs underwent a Y-maze test. After the behavioral experiment, animals administered with PGE (root) were sacrificed and brains were isolated for analysis. Subsequently, immunohistochemistry of brains was performed using DCX antibody and immunoblotting with SYN antibody. PGE, *Platycodon grandiflorus* extract. DCX, doublecortin. SYN, synaptophysin.

All male C57Bl/6 mice were eight weeks of age and housed under standard laboratory conditions. Animals were treated in accordance with the National Institutes of Health guide for the care and use of Laboratory animals (NIH Publications No. 8023, revised 1978) and under the supervision of the Institutional Animal Care and Use Committee at Konyang University. The mice were randomly divided into four groups (*n* = 10–12 in each group): (1) PGE (root) group, administered with the PGE (root); (2) PGE (aerial part) group, administered with the PGE (aerial part); (3) PGE (seed) group, administered with the PGE (seed); and (4) control group, administered with phosphate-buffered saline (PBS). PGEs (100 mg/kg/day) were administered orally by gavage needle once a day for 15 consecutive days. A cognitive behavioral test was performed on the 16th day of PGE treatment before sacrifice (17 days after the first treatment of PGE) (Figure 1).

### 2.7. Brain Tissue Preparation

Following the last behavioral test, animals were immediately anesthetized and transcardially perfused with 0.05 M PBS, and then fixed with cold 4% paraformaldehyde (PFA) in 0.1 M phosphate buffer. Brain tissues were removed and post-fixed in 0.1 M phosphate buffer containing 4% PFA overnight at 4 °C and then immersed in a solution containing 30% sucrose in 0.05 M PBS for cryoprotection. Serial coronal sections (30 µm-thick) were cut on a cryostat microtome and stored in a cryoprotectant (25% ethylene glycol, 25% glycerol, and 0.05 M phosphate buffer) at 4 °C until use for immunohistochemistry analysis.

### 2.8. Immunohistochemistry Analysis and Quantification

For immunohistochemical analysis, brain sections were rinsed briefly in PBS and treated with 1% hydrogen peroxide for 15 min. The sections were incubated with goat anti-doublecortin (DCX) antibody (1:1000) overnight at 4 °C. The sections were then incubated with biotinylated horse anti-goat IgG (1:200) and avidin-biotin-peroxidase complex solution, and then visualized using a SIGMA FAST™ 3.3’-diaminobenzidine tablet (Sigma-Aldrich) as a chromogen. To quantify immunoreactivity, the images were processed and analyzed using Image-Pro Plus 6.0 software (Media Cybernetics, Bethesda, Rockville, MD, USA). The analysis was performed blindly in both hemispheres of 5–6 brain sections per animal. The number of DCX-positive cells in the DG were counted using Image-Pro Plus Version 6.0 (Media Cybernetics, Inc., Rockville, MD, USA).

### 2.9. Western Blot Analysis

Tissues from the frontal cortex and hippocampus of mouse brains were lysed using a protease inhibitor cocktail (Roche, Mannheim, Germany), with the addition of a triple-detergent lysis buffer (50 mM Tris-HCl, 150 mM NaCl, 0.1% SDS, 1% NP-40). Protein concentrations in lysates were quantified by the Bradford assay (Sigma-Aldrich). The lysates were separated by 12% sodium dodecyl sulfate-polyacrylamide gel electrophoresis (SDS-PAGE) and then transferred to a nitrocellulose membrane, 0.45 µm (Bio-Rad, Hercules, CA, USA). The membranes were incubated with 5% skim milk in Tris–buffered saline with Tween 20 (TBST) for 1 h and then with a primary antibody (1:1000 dilution for synaptophysin; 1:5000 dilution for β-actin) overnight at 4 °C. This was followed by incubation with a horseradish peroxidase conjugated secondary antibody for 1 h. Immunoreactive-bands were detected using a chemiluminescent substrate (Thermo Fisher Scientific, Waltham, MA, USA). Band intensities were normalized to the β-actin band intensity using ImageJ software (National Institutes of Health (NIH), Bethesda, MD, USA).

### 2.10. Y-Maze Test

The Y-maze task was performed according to a method described previously [47]. The Y-maze apparatus has three arms separated by 120° angles (30 cm long × 8 cm wide × 15 cm high) extending from a central space (8 × 8 cm). Each mouse was placed in one arm and allowed to explore freely for 5 min to assess its rate of spontaneous alternation. Spontaneous alternation is defined as successive entries into three different arms consecutively without repetition (i.e., ABC, BCA, but not ABA). The spontaneous alternation percentage was calculated by the following equation: (successive entries/(total arm entries − 2) × 100).

### 2.11. Cell Viability Assay

A MTT (3-(4,5-dimethylthiazol-2yl)-2,5-diphenyltetrazolium bromide) assay was conducted to examine the effects of PGE (root) on cell viability. PC12 cells (5 × 10^3^ cells/well) were placed in single cell suspensions and seeded in individual wells of 96-well plates prior to exposure to culture plates containing 0, 10, 50, 100, 250, 500, and 1000 μg/mL of PGE (root) for 24 h at 37 °C. MTT solution was added to each well followed by incubation for 4 h at 37 °C prior to removing the culture medium. DMSO was then added and mixed for 30 min at room temperature. Cell viability was determined by measuring the absorbance at 562 nm. The cell viability for each group was calculated as a percentage of that of the control group.

### 2.12. PC12 Cell Culture and Treatment of PGE, Platycodin D, and Platycodin D2

PC12 cells were cultured in Dulbecco’s Modified Eagle’s Medium (DMEM) with 5% fetal bovine serum, 10% horse serum (HS), and 1% penicillin-streptomycin (PS), and were maintained in a 5% CO_2_ incubator at 37 °C. PC12 cells (1 × 10^5^ cells/well) were seeded into six-well poly-d-lysine-coated plates. Medium was refreshed after 24 h. Three separate experiments were performed to investigate the effects of PGE (root) and the bioactive constituents of PG. PC12 cells were treated with PGE (root) (10 μg/mL), platycodin D (10 μg/mL), or platycodin D2 (10 μg/mL) in DMEM medium containing 1% fetal bovine serum, 2% HS, and 1% PS.

### 2.13. Quantification of Neurite Outgrowth of PC12 Cells

To evaluate the effects of PGE (root) and single constituents of PG root on neurite outgrowth, the lengths of PC12 neurites were measured after treatment with PGE (root) or bioactive molecules; platycodin D and D2. After 24 h, images of neurite outgrowths were obtained from 10 random fields of each cell group. The collected images of neurites were used to determine the lengths of neurites using ImageJ (NIH). 

### 2.14. Statistical Analysis

All data are shown as the mean ± standard error of the mean (SEM). The statistical differences of variables between two groups were analyzed by an independent T-test and differences among the four groups were analyzed by one-way ANOVA, followed by Fisher’s LSD post hoc test using SigmaStat for Windows Version 3.10 (Systat Software, Inc., Point Richmond, CA, USA). A *p*-value < 0.05 was considered statistically significant.

## 3. Results

### 3.1. Behavioral Tests in Mice to Determine Cognitive Function after Administration of PGEs

**Figure 2:**
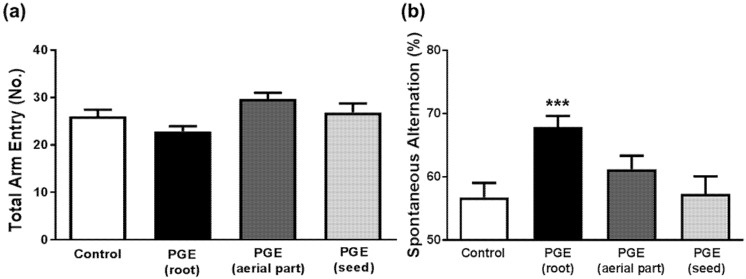
Behavioral test to determine the enhancing effect of PGE on cognition. The Y-maze test shows no significant influence of PGE on total arm entry regardless of the PG structural part administered (**a**). The PGE (root)-administered group shows a significant increase in spontaneous alternations, indicating PGE (root) significantly increased spatial learning and memory. PGE (aerial part) and PGE (seed)-administered groups show no significant increase of spontaneous alternation (**b**). Values are the mean + SEM. *** *p* < 0.05 compared to the vehicle-treated control group. PGE, *Platycodon grandiflorus* extract.

Previous studies have shown that the root of PG ameliorates memory impairment [36,38,39] and improves cognitive function [37,40], while the effects of other parts (aerial part and seeds) were not examined. To investigate whether the root of PG, as well as the aerial part and seeds of PG, mediate its memory-enhancing effects, respectively, and to compare the effects of three different parts of PG, we carried out a behavioral test with mice administered different parts of PG. PGEs had no significant influence on locomotor activity, as shown in total arm entry (Figure 2a). However, administration of PGE (root) significantly increased spontaneous alternations in the Y-maze test. Interestingly, there was no significant difference between PGE (aerial part) or PGE (seed) groups and the control group (Figure 2b). These results indicate that only root part has memory-enhancing effect but other parts (aerial part and seed) do not have any effect on spatial working memory in mice.

### 3.2. Standardization of Platycodin D and Platycodin D2 in PGE (Root) Using HPLC

**Figure 3:**
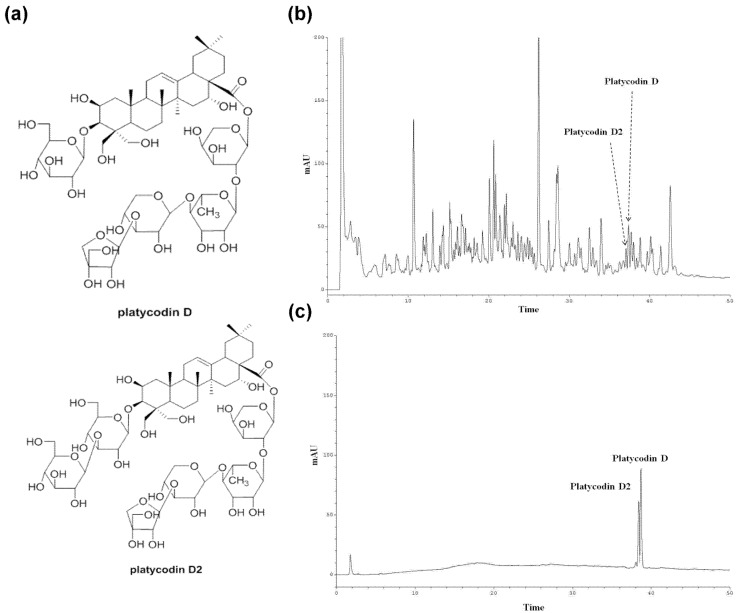
HPLC chromatograms of the EtOH extract of PG. (**a**) Structures of platycodin D and platycodin D2, (**b**) HPLC chromatograms of the root extract, and (**c**) HPLC chromatograms of standard compounds, platycodin D, and platycodin D2. HPLC analysis was carried out using a gradient elution on a phenomenex Kinetex RP-C18 column (5 µ, 100 A, 150 × 4.6 mm) at a flow rate of 1.0 mL/min, and with UV 210 nm detection.

Contents of platycodin D and platycodin D2 were determined by HPLC analysis with a reverse phase C-18 column. The HPLC chromatogram of the extract was prepared for the PGE (root), two standard compounds were observed in its HPLC chromatogram with UV 210 nm detection (Figure 3b,c). Platycodin D and platycodin D2 were isolated from *n*-butanol fraction of the root extract by means of chromatographic separation and purification (Figure 3a). These compounds were identified by comparing their spectral data (Figure S1) with literature values [48]. Using standard curves of the compounds, contents of platycodin D and platycodin D2 in the PGE (root) were estimated as 2.75 and 1.50 μg/mg, respectively.

### 3.3. Immunohistochemical Analysis of the effect of PGE (root) on Hippocampal Neurogenesis

**Figure 4:**
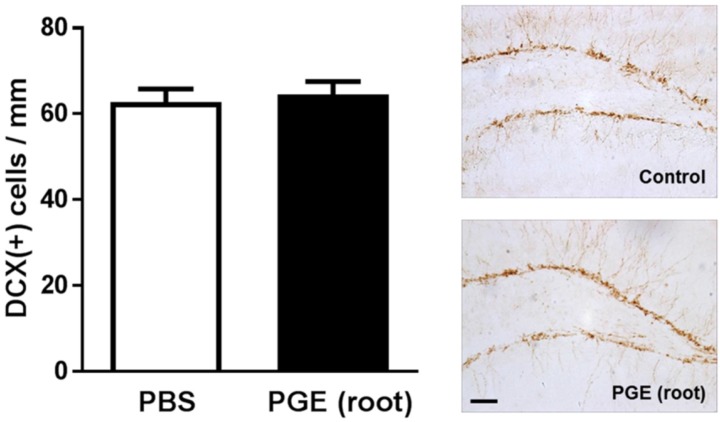
Immunohistochemical analysis using DCX antibody. DCX is a marker of neurogenesis, especially neuronal fate specification. The PGE (root)-administered group shows a similar number of DCX (+) cells per unit length compared to the control group. Values are the mean + SEM. Scale bar = 50 μm. PGE, *Platycodon grandiflorus* extract. DCX, doublecortin.

Previous biochemical studies demonstrated that PGE may improve learning and memory by inhibiting acetylcholinesterase activity [39]. However, no evidence of histological changes induced by PG during the enhancement of cognitive function has been reported. Interestingly, PG root was shown to significantly induce adult neurogenesis in the hippocampus of middle-aged mice [19]. Thus, to investigate the histological mechanism of improved cognitive function by PGE-administration, we performed immunohistochemistry using a common neurogenesis marker, DCX. However, as shown in Figure 4, there was no significant difference in the number of DCX (+) cells between the control and PGE (root) groups. Therefore, our finding suggests that PGE-induced enhancement of cognition is not mediated by the induction of adult hippocampal neurogenesis in young mice.

### 3.4. Immunoblotting Analysis of the Effect of PGE (root) on Synaptic Sprouting

**Figure 5:**
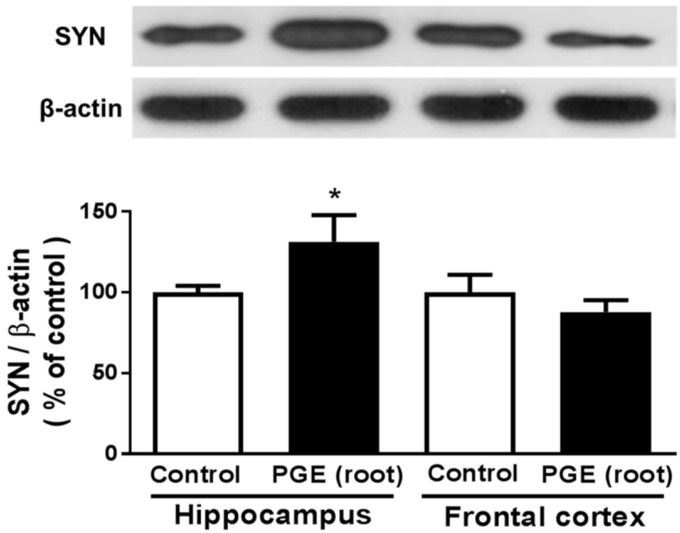
Immunoblotting analysis of synaptophysin expression in the hippocampus and frontal cortex after PGE (root)-administration. The PGE (root)-administered group shows a significantly higher expression of SYN in hippocampus than the controls. The PGE (root)-administered group shows no significant difference in the frontal cortex compared to the controls. Values are the mean + SEM. * *p <* 0.05 compared to the vehicle-treated control group. PGE, *Platycodon grandiflorus* extract. SYN, synaptophysin.

To investigate the mechanism of cognitive improvement by PG root-administration, we performed immunoblotting with synaptophysin, a molecular marker of synaptogenesis [49]. It was reported that the prefrontal cortex and hippocampus are important for spatial working memory [50]. The PGE (root) group showed a significant increase in synaptophysin expression in the hippocampus, but not in the frontal cortex, compared with the control group (Figure 5). These findings demonstrate that the PGE (root)-induced enhancement of memory or cognition may be mediated by increased synaptogenesis in the hippocampus.

### 3.5. PGE (Root) Increases Neuritogenesis In Vitro

**Figure 6:**
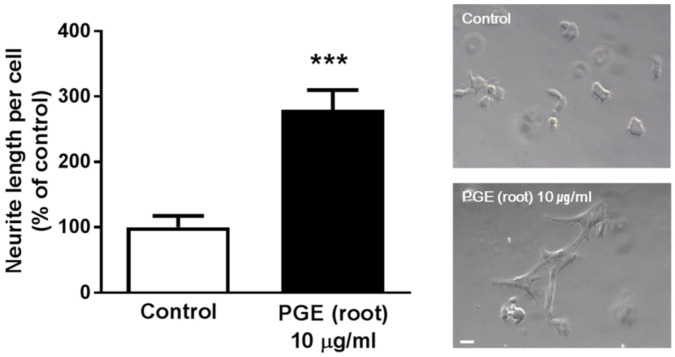
In vitro assay to confirm PGE (root)-induced neuritogenesis in PC12 cells. PC12 cells were treated with 10 μg/mL PGE (root) for 24 h and the cells show a significant increase in neurite length compared to the controls. Values are the mean + SEM. *** *p <* 0.05 compared to the vehicle-treated control group. Scale bar = 20 μm. PGE, *Platycodon grandiflorus* extract.

To investigate whether PGE can directly influence synaptogenesis in a pure neuronal culture, we carried out in vitro experiments with PC12 cells to confirm the effect of PGE (root) on neuritogenesis, the extension of neurites [51]. As PC 12 cells were not affected by up to 10 μg/mL of PGE (root) (Figure S2), PC12 cells were treated with PGE root (10 μg/mL) for 24 h and observed for morphological changes. PGE (root) significantly increased the neurite length outgrowth by 200% compared to the control group (Figure 6). This result shows that PGE (root) directly induces neuritogenesis in neuronal cells.

### 3.6. PGE (Root) Activates the MAPK/ERK Signaling Pathway

**Figure 7:**
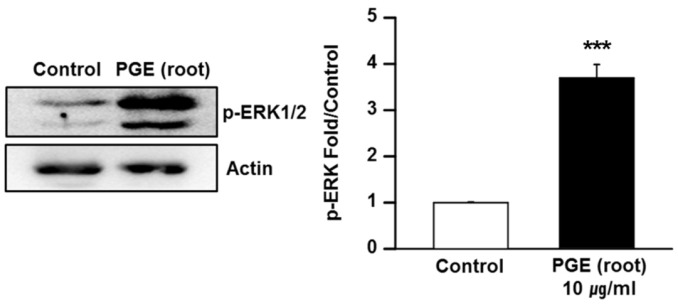
Immunoblotting of PGE (root) treated PC12 cells with ERK1/2. Cells were treated with PGE (root) for 24 h and assayed by western blot using anti-ERK1/2 antibodies and anti-phospho-ERK1/2 antibodies (Thr202/Tyr204). PGE-(root) treated PC12 cells show a significant increase in ERK1/2 phosphorylation. Values are the mean + SEM. *** *p <* 0.05 compared to the vehicle-treated control group. PGE, *Platycodon grandiflorus* extract.

It has been demonstrated that PGE activates the MAPK/ERK signaling pathway in several cell lines [42]. To elucidate the mechanism by which PGE administration improves cognitive function, we conducted in vitro experiments with PC12 cells to determine changes in the phosphorylation of ERK1/2, which is a key downstream component of the MAPK/ERK pathway. PC12 cells were treated with 10 μg/mL of PGE (root) for 24 h, and both total and phosphorylated ERK1/2 levels were examined by Western blot analysis. As shown in Figure 7, after 24 h treatment with PG root, there was no difference in the level of total ERK1/2 between the PGE (root)-treated cells and controls. However, phosphorylation of ERK1/2 was markedly increased in PGE-treated cells. This in vitro data indicates that the neuritogenic effect of PGE (root) may be mediated by the activation of MAPK/ERK signaling cascades.

### 3.7. Platycodin D, a Bioactive Ingredient of PG, Increases Neuritogenesis In Vitro

**Figure 8:**
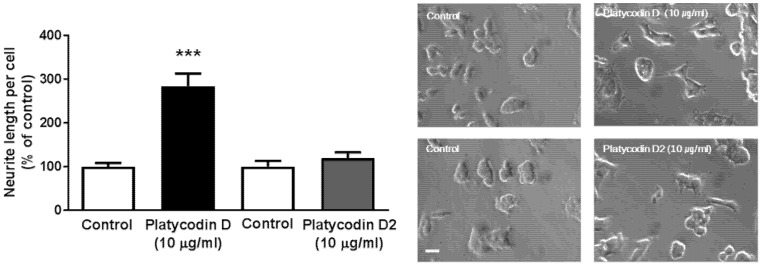
Measurement of neurite length to confirms the effects of bioactive molecules of PG in PC12 cells. PC12 cells were treated with 10 μg/mL platycodin D or platycodin D2 for 24 h and neurite lengths measured. Platycodin D, but not platycodin D2, treated PC12 cells show significantly increased neurite lengths. Scale bar = 20 μm. Values are the mean + SEM. *** *p <* 0.05 compared to the vehicle-treated control group.

The characterization of bioactive constituents is necessary to understand the effects of natural products, and chromatographic analyses are widely used to examine the bioactive ingredients from natural products [52]. We investigated the neuritogenic effects of platycodin D and platycodin D2, which are bioactive molecules of PG [28,42,53], in PC12 cells. To investigate the effects of platycodin D and platycodin D2 on neuritogenesis, we evaluated the neurite extension of PC12 cells. Following 24 h of platycodin D and platycodin D2 treatment (10 μg/mL), we observed a significant increase in neurite outgrowths in platycodin D-treated cells compared to controls (Figure 8). However, there was no significant change in the length of neurites in platycodin D2-treated PC12 cells (Figure 8). This strongly suggests that platycodin D, a bioactive constituent of PG, has a stimulating effect on neuritogenesis during memory formation.

## 4. Discussion

To examine whether the root of PG, as well as aerial part and seeds of PG, improve cognitive function and to uncover the underlying mechanisms of this effect might be important for the development of potential therapeutic agents to enhance cognitive functions. However, studies to identify the specific parts and constituents of PG associated with memory-enhancing activity in animals have not been reported. In the present study, we found that PG root induced a cognitive improvement in mice. The neurostructural and molecular mechanisms underlying the PG-mediated improvement of cognition were examined by assessing hippocampal neurogenesis, axonal outgrowth, neuritogenesis, and activation of the ERK signaling pathway. Administration of PGE (root) extract significantly increased synaptogenesis, but not adult hippocampal neurogenesis, as demonstrated by the enhanced expression of synaptophysin, a synaptogenic marker, in the hippocampus of mice. In addition, PG-treated PC12 cells showed increased neuritogenesis and the upregulation of phosphorylated ERK1/2. These data suggest that the PG root enhances cognitive function through ERK activation and synaptogenesis in the hippocampus.

In the behavioral test using a Y-maze, we examined which parts of PG improved cognitive function in three different groups treated with three different PG parts (root, aerial part, and seeds). The result of the Y-maze task indicated that PG root, but not the aerial part or seeds, had a potent stimulating effect on cognitive enhancement. Based on the lack of effect on cognitive functions of the aerial part and seeds, it can be speculated that PG displays structure-specific effects on memory functions.

Many previous studies have demonstrated that PG root improves cognitive functions in animal models of memory impairment. In a mouse model of amnesia induced by EtOH treatment, the oral administration of whole PG root extract, including platycodin D and platycoside E, prolonged step through latency (STL) in a passive avoidance test (PAT), suggesting that PG may inhibit memory deficits [38]. Similarly, PGE and platycodon extract jelly reduced escape latency time in a Morris water maze and prolonged STL in a PAT in cognition-impaired mice induced by scopolamine [37]. PGE also exhibited protective effects on neuronal loss induced by Aβ, which is the main component of amyloid plaques that leads to synaptic loss in Alzheimer’s disease (AD). In vitro experiments with PC12 cells showed that Aβ-mediated cell death was inhibited by the administration of PGE [37]. Moreover, through the free radical scavenging effect and anticholinesterase inhibition properties of PG, the PGE (root) and its fermented production showed cognitive-enhancing effects in the PAT, Y-maze, and Morris water maze in a mouse model of scopolamine-induced amnesia [54]. These data suggest that the antioxidant, anticholinesterase, and antitoxic effects of PG root might contribute to the amelioration of cognitive impairment in animal models of impaired memory. In accordance with the aerial parts and seeds did not affect memory functions, previous study indicated that platycodin D, the bioactive ingredient inducing neurite outgrowth was not found in the aerial parts [46], as well as found approximately eight times more in the root than in other parts, such as stems and leaves [43]. Therefore, the observed results in behavioral test may also be explained by differences of contents among the parts of PG. Additionally, supplementary behavioral tests might be advantageous for evaluating the effects of PG on various types of memories.

To date, the mechanisms underlying the cognitive improving effects of PG have not been clearly explained. Thus, we investigated these mechanisms of PGE (root)-mediated behavioral changes by using both histological and molecular approaches. We performed immunohistochemistry to determine whether the induction of adult hippocampal neurogenesis or synaptogenesis may mediate PGE (root)-induced memory enhancement. A previous study reported that PG might stimulate adult neurogenesis in the hippocampus of middle-aged mice (12 months) [19], we did not observe an increase in neurogenesis caused by PGE (root) administration during the enhancement of cognition in mice. Although this result may contradict the previous report, it can be speculated that different treatment regimen, dose, and number of daily administrations might have affected hippocampal neurogenesis differently. Moreover, the discrepancy between our findings and those of the previous study might be explained by differences in the age of animals and extraction methods used. Since adult neurogenesis is highly age-dependent, the difference in mice ages between our study (eight weeks) and the previous study (12 months) [19] may explain this discrepancy at the level of DCX-positive neurons. It was reported that adult hippocampal neurogenesis is gradually decreased by approximately 40% in two-month old mice and 95% in 10-month old mice compared to one-month old C57Bl/6J mice, respectively [55]. Considering that adult neurogenesis has a dependence on age, adult neurogenesis in control mice might still have been maintained enough to mask the increased adult neurogenesis of experimental mice induced by PGE administration. This might lead to the adult neurogenesis not being significantly increased in PGE (root)-administered mice at the age of two=-months. In addition, a discrepancy between our findings and a previous study imply that PG may have stimulating effects on adult neurogenesis more in middle-aged mice than young adult mice. Further studies are needed to confirm the age-dependent effects of PG on adult hippocampal neurogenesis in mice.

Using a pure neuronal culture, we found that PGE (root) and its bioactive ingredients directly stimulated neurite outgrowth in PC 12 cells (Figure 6 and Figure 8). Few studies have reported the signaling pathways involved in the induction of synaptogenesis by PG treatment. It has been reported that PG treatment increased activation of the ERK signaling pathway in osteoblasts and endothelial cells [42]. Interestingly, we observed the activating action of PG on both neurite outgrowth and ERK signaling pathway in neuronal cells (Figure 7). A large number of previous studies have shown the stimulating effects of ERK1/2 and natural products from plants on neurite outgrowth [56,57,58,59,60]. It has been reported that ginsenoside Rg1 and Rb1, and triterpenoid saponins, enhance the neurite outgrowth of hippocampal neurons by activating the ERK1/2 and PI3K/Akt pathways [56,57]. Ginsenoside Rd treatment also promotes the neurite outgrowth of PC12 cells by activating the MAPK/ERK and PI3K/Akt signaling proteins [60]. In human endothelial cells, pretreatment with ginsenoside Rg1 up-regulated glucocorticoid receptor dependent ERK phosphorylation [58]. Furthermore, another triterpenoid saponin, Chikusetsu saponin IVa protected cardiomyocytes from hyperglycemic-triggered oxidative stress by activation of ERK1/2 signaling proteins [59]. Based on previous reports and our current findings, we speculate that upregulated ERK1/2 phosphorylation by PG treatment may, in part, contribute to cognitive enhancement by increasing synaptogenesis in the hippocampus.

Nevertheless, an additional possibility that non-neuronal cells, such as microglia, astrocytes, and endothelial cells, can also affect hippocampal synaptogenesis might be raised by previous studies reporting the protective effects of the root of PG against oxidative stress in the glioma cells [61] and inflammatory responses in microglial cells [34]. Furthermore, the present study is the first to examine the molecular signaling pathways underlying the PGE (root)-mediated enhancement of learning and memory, there might be a large number of signaling pathways involved in the effects of PGE (root) treatment. Thus, further study is needed to identify which signaling molecules are related to hippocampal synaptogenesis induced by PGE (root) treatment. Additionally, use of memory-impaired animal models, such as animals carrying familial AD (FAD) mutant genes showing similar cognitive features of AD, would be useful for understanding therapeutic efficacy, as well as the beneficial effects of PGE (root) on neurodegenerative diseases. 

## 5. Conclusions

Taken together, the present study demonstrated that PGE (root), containing platycodin D, enhanced cognitive function in mice through increased synaptogenesis in the hippocampus which is, in part, mediated by activation of the MAPK/ERK pathway. The present study also describes the mechanisms that underlie the cognitive enhancing effects of PGE (root). Therefore, we suggest that PGE (root) might be a potential candidate treatment of memory disorders through its synaptogenesis-promoting effects in the hippocampus.

## Supplementary Materials

The following are available online at www.mdpi.com/2072-6643/9/7/794/s1, **Figure S1.** NMR spectroscopy data of platycodin D and platycodin D2; **Figure S2.** Cell viability of PC12 cells under PGE treatment.
