# Supplementary material for: Platycodon grandiflorus Root Extract Improves Learning and Memory by Enhancing Synaptogenesis in Mice Hippocampus
Source: Nutrients. 2017 Jul 23;9(7):794. doi: 10.3390/nu9070794 (PMC5537907; doi:10.3390/nu9070794)
Supplement: Supplementary file 1 [file nutrients-09-00794-s001.zip › Supplementary material.docx]

**Supplementary Materials: *Platycodon grandiflorus* root extract improves learning and memory by enhancing synaptogenesis in mice hippocampus**

Jin-il Kim, Seong Gak Jeon, Kyoung Ah Kim, Jwa-Jin Kim, Eun Ji Song, Yukyoung Jeon, Eunbin Kim, Kyung Bok Lee, Jong Hwan Kwak and Minho Moon


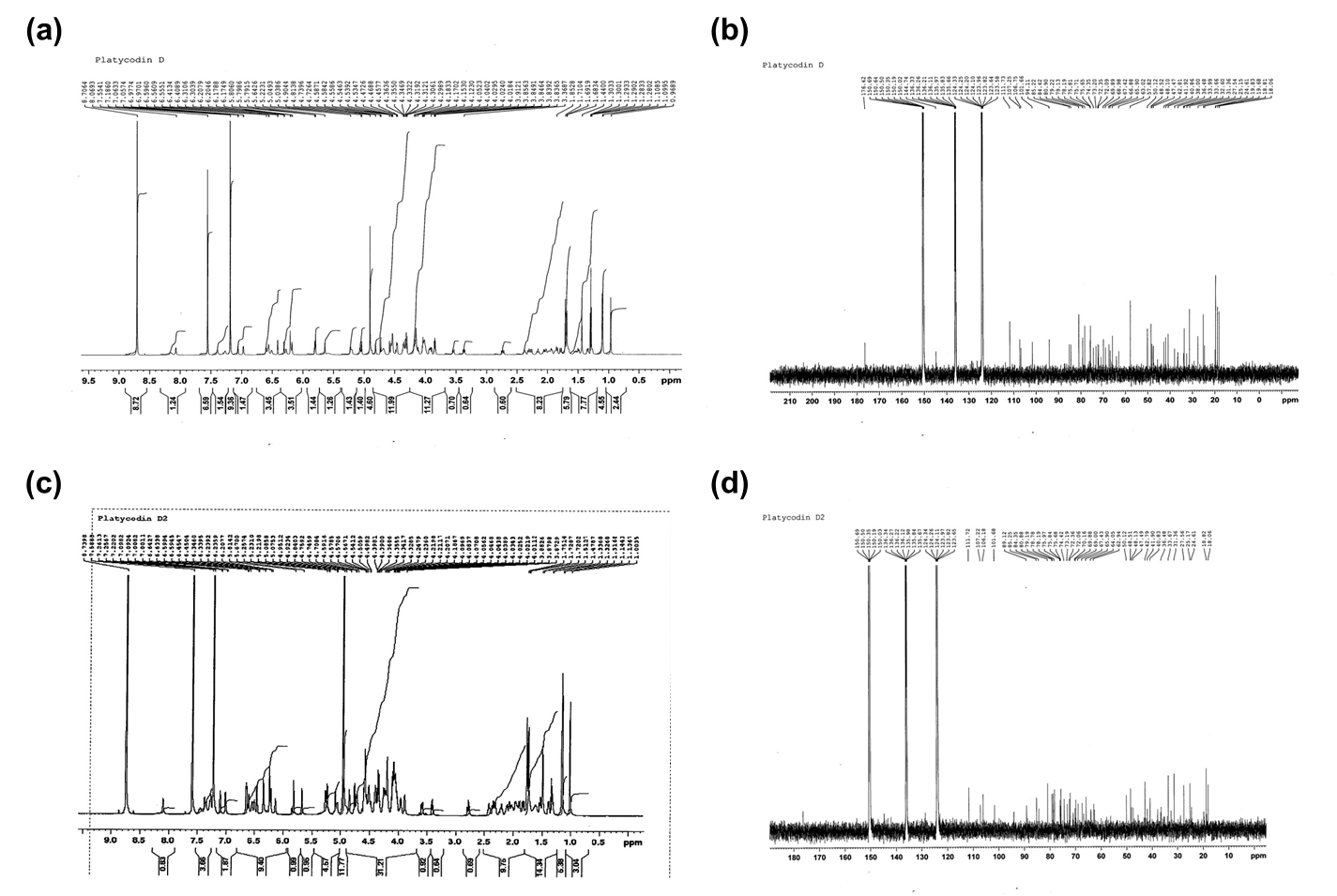


**Figure S1.** NMR spectroscopy data of platycodin D and platycodin D2. NMR experiments were performed on a Bruker AVANCE Ⅲ 700 spectrometer with the usual pulse sequence, and chemical shifts (*δ*) are reported in parts per million, referenced to the solvent used. ESI-MS data was obtained on a Waters Acuity Ultra Performance LC-MS system LCA 048 (Waters, USA). Column chromatography was performed on silica gel 60 (230-400 mesh, Merck, Darmstadt, Germany), LiChroprep RP-18 (40-63 ㎛, Merck), and Sephadex LH-20 (25-100 μ, Sigma-Aldrich, USA). (a) ^1^H-NMR spectrum of platycodin D (700 MHz, pyridine-*d_5_*), (b) ^13^C-NMR spectrum of platycodin D (176 MHz, pyridine-*d_5_*), (c) ^1^H-NMR spectrum of platycodin D2 (700 MHz, pyridine-*d_5_*), (d) ^13^C-NMR spectrum of platycodin D2 (176 MHz, pyridine-*d_5_*).

0

50

100

150

**Cell viability (%)**

C

1000

500

250

(ug/ml)

**PGE (root)**

75

25

125

100

50

10

*****

*****

*****

*****

*****

**Figure S2.** Cell viability of PC12 cells under PGE treatment. PC12 cells were cultured with different doses of PGE for 24 hours. MTT assay was performed to determine the cell viability. Values are the mean + SEM. **p*<0.05 compared to the vehicle-treated control group. MTT, 3**-**(4,5-dimethylthiazol-2yl)-2,5**-**diphenyltetrazolium bromide. PGE, *Platycodon grandiflorus* extract.
